# Analysis of *pir* gene expression across the *Plasmodium* life cycle

**DOI:** 10.1186/s12936-021-03979-6

**Published:** 2021-11-25

**Authors:** Timothy S. Little, Deirdre A. Cunningham, Audrey Vandomme, Carlos Talavera Lopez, Sarah Amis, Christopher Alder, John W. G. Addy, Sarah McLaughlin, Caroline Hosking, George Christophides, Adam J. Reid, Jean Langhorne

**Affiliations:** 1 The Francis Crick Institute, London, UK; 2 Imperial College, London, UK; 3 Wellcome Sanger Institute, Cambridge, CB10 1SA UK; 4 Present Address: Institute of Computational Biology, Helmholtz Zentrum für Gesundheit und Umwelt, Munich, Germany

## Abstract

**Background:**

*Plasmodium* interspersed repeat (*pir*) is the largest multigene family in the genomes of most *Plasmodium* species. A variety of functions for the PIR proteins which they encode have been proposed, including antigenic variation, immune evasion, sequestration and rosetting. However, direct evidence for these is lacking. The repetitive nature of the family has made it difficult to determine function experimentally. However, there has been some success in using gene expression studies to suggest roles for some members in virulence and chronic infection.

**Methods:**

Here *pir* gene expression was examined across the life cycle of *Plasmodium berghei* using publicly available RNAseq data-sets, and at high resolution in the intraerythrocytic development cycle using new data from *Plasmodium chabaudi*.

**Results:**

Expression of *pir* genes is greatest in stages of the parasite which invade and reside in red blood cells. The marked exception is that liver merozoites and male gametocytes produce a very large number of *pir* gene transcripts, notably compared to female gametocytes, which produce relatively few. Within the asexual blood stages different subfamilies peak at different times, suggesting further functional distinctions. Representing a subfamily of its own, the highly conserved ancestral *pir* gene warrants further investigation due to its potential tractability for functional investigation*.* It is highly transcribed in multiple life cycle stages and across most studied *Plasmodium* species and thus is likely to play an important role in parasite biology.

**Conclusions:**

The identification of distinct expression patterns for different *pir* genes and subfamilies is likely to provide a basis for the design of future experiments to uncover their function.

**Supplementary Information:**

The online version contains supplementary material available at 10.1186/s12936-021-03979-6.

## Background

The genomes of the malaria parasites (*Plasmodium* spp.) contain a variety of multigene families. These are generally located in the sub-telomeric regions of chromosomes, a feature which is thought to allow regulation of gene expression by heterochromatin and promote diversification of the gene sequences through recombination [1–3]. The most highly studied of these families is *var*, which encodes the PfEMP1 protein. It is present in ~ 70 copies in the human malaria parasite *Plasmodium falciparum*. The primary role of PfEMP1 appears to be sequestration of the parasite in the vasculature, preventing its destruction in the spleen [4]. Multiple copies of *var* seem to be required to alter the binding specificity and for immune evasion—i.e. switching between antigenically distinct proteins with similar binding properties [5]. This family is however limited to the *Laverania* subgenus, which includes only one of the five species which infect humans. The *Plasmodium* interspersed repeat (*pir*) multigene family has been found in the genomes of rodent malarias, primate malarias and the human-infecting *Plasmodium* species, *Plasmodium vivax*, *Plasmodium knowlesi*, *Plasmodium malariae* and *Plasmodium ovale* [6–9]. The number of *pir* genes in the genomes of these species varies considerably, from the lower numbers of 211 members in *Plasmodium chabaudi chabaudi* (AS strain) and 134 members in *Plasmodium berghei*, to more than 1,000 members in *Plasmodium yoelii* and up to 1,949 members in *P. ovale curtisi* [9, 10]. The *pir* family is, therefore, considered to be the largest *Plasmodium* multigene family.

Although it has been suggested that the *pir* family fulfils similar roles in immune evasion and pathogenesis as *P. falciparum var*, there is no direct evidence for this, and the function(s) of *pir*s remains largely unknown. While *P. falciparum* parasites can only express a single *var* gene at a time, individual *P. berghei, P. vivax* and *P. yoelii* parasites can express multiple *pir* genes [11–15]. Although some proteomic and immunofluorescence studies show PIR proteins on the surface of infected RBCs (iRBCs), other studies indicate that they are present in the host or parasite cytoplasm, or on the parasitophorous vacuole [6, 16–19] suggesting multiple different functions for PIR proteins during blood-stage infections. In *P. c. chabaudi* (AS strain) infections of mice, different *pir* subfamilies are associated with the acute and chronic phases of the infection, and parasites from the two phases of infection are differently virulent [20]. This association suggests *pir* gene expression may affect virulence of blood-stage *P. chabaudi*, and also that *pir* genes could be involved in evading the initial immune response. Single-cell RNA-seq analysis across the *P. berghei* life cycle has shown that *pir* gene expression is particularly high in blood stages [14]. Male gametocytes were found to express a distinct *pir* repertoire compared to asexual blood stages, with female gametocytes expressing few *pir* transcripts [13]. Although these data represent only the most highly expressed genes, they suggest there may be different functions for *pir* genes between asexual and sexual blood stages.

This family has been very difficult to study in the laboratory due to there being many genes with varying levels of similarity expressed at a variety of times. Identifying which are expressed at particular life-cycle stages will allow a more targeted approach to determining function. Here two rodent-infecting *Plasmodium* species were used to investigate the stage-specificity of *pir* gene expression. A systematic analysis of the *P. berghei* (ANKA strain) *pir* multigene family from published bulk RNA-seq studies was performed, re-processing the raw data using a single bioinformatics pipeline. While single-cell RNA-seq datasets have provided excellent resolution of gene expression in time across the life cycle [14], bulk RNA-seq datasets provide much higher resolution of the transcriptome itself, detecting a larger proportion of transcripts expressed at any one time. Indeed, expression of relatively few *pir* genes was detected in the Malaria Cell Atlas [14]. To examine the dynamics of *pir* expression in asexual blood stages in more detail, high-resolution transcriptional data was generated from *P. c. chabaudi* (AS strain), across the asexual blood cycle.

This study demonstrates that the primary function of the *pir* multigene family likely plays out in the blood stages, from merozoite formation in the liver to fertilization in the mosquito midgut. It is unlikely to be important during the post-fertilization stages of development in the mosquito through to early liver stages. In both rodent malaria models, the *pir* transcriptional repertoire is diverse throughout the intraerythrocytic developmental cycle, however different subfamilies are differently represented over time, suggesting subtle differences in regulation and perhaps function. One *pir* gene, previously described as the putative ancestral *pir* [21], is clearly notable as forming the highest proportion of the *pir* transcriptome of these two species, and of multiple different species of *Plasmodium* for which transcription data is available. This gene may prove a more experimentally tractable target due to its uniqueness and warrants further study.

## Methods

### Mice

Female C57BL/6 aged 6–8 weeks from the SPF unit at the Francis Crick Institute Mill Hill Laboratory were housed under normal (light 07.00–19.00, dark 19.00–07.00 GMT) or reverse light conditions (light 19.00–07.00, dark 07.00–19.00 GMT) at 20–22 °C, and had continuous access to mouse breeder diet and water. This study was carried out in accordance with the UK Animals (Scientific Procedures) Act 1986 (Home Office licence 80/2538 and 70/8326) and was approved by The Francis Crick Institute Ethical Committee.

### Parasites

A cryopreserved stock of a cloned line of *Plasmodium chabaudi chabaudi* (AS strain), originally obtained from David Walliker, University of Edinburgh, UK, and subsequently passaged through mice by injection of infected red blood cells (iRBC), was used to initiate infections and mosquito transmissions. Transmission of *P. chabaudi* via *Anopheles stephensi* mosquitoes has been described in detail previously [22]. Recently mosquito-transmitted (RMT) *P. chabaudi* derived from a mosquito-initiated infection retain the same phenotype of infection course and gene expression profiles of directly mosquito-transmitted *P. chabaudi* infections [20, 23]. RMT parasites are used here to ensure that each mouse receives a consistent inoculum of blood-stage parasites, rather than the temporarily more variable appearance of parasites in the blood from either a MT- or sporozoite-initiated infection [23]. For RMT-blood stage infections, mice were infected by intraperitoneal injection of 10^5^ iRBC. Blood samples were collected at three-hour intervals over one 24 h asexual cycle. Infections were monitored by light microscopy on Giemsa-stained thin blood smears. Parasitaemia across the 24 h ranged from 2 to 10% (Additional file 6).

### RNA extraction and sequencing

Mice infected as described above were segregated into eight groups (3 mice/group) according to the scheduled time points 02:00; 05:00, 08:00, 11:00, 14:00, 17:00, 20:00, 23:00 and at each time point, day 7 post-infection, a group was smeared, exsanguinated and blood collected for RNA extraction. Blood samples were depleted of leukocytes by filtration (Plasmodipur, Euro Proxima) and of globin RNA by saponin lysis and centrifugation and RNA extracted as previously described [23]. Briefly, purified parasites were resuspended in Trizol, frozen at – 80 °C until use. RNA was then extracted and resuspended in water.

*P. chabaudi* RNA samples were used to make 150–350 bp fragment Illumina TruSeq libraries, with 12 cycles of PCR amplification. All 24 samples were tagged, pooled and sequenced on each of two Illumina HiSeq2500 lanes with paired 100 bp reads.

### RNAseq analysis

RNAseq datasets were downloaded from SRA using SRA-Tools (SRA_Toolkit_Development_Team), except for the *P. chabaudi* data and the *P. berghei* data from Witmer et al. [24] and Ukegbu et al. [25], which was transferred manually from the original lab data storage. Technical replicates were concatenated together as fastq.gz files. Both the *P. berghei* and the *P. chabaudi* RNAseq were processed through the rna-seq pipeline v1.4.2 from nf-core repository in the Nextflow pipeline software v20.07.1 [26, 27], using Trim Galore! v0.6.4 to trim sequences, HISAT2 v.2.1.0 as the aligner, and featurecounts v.1.6.4 as the gene count program [28–30]. Quality control was analysed using FastQC v.0.11.8 and collated by MultiQC v.1.7 [31, 32]. Paired-end sequence files and single-end sequence files for *P. berghei* were separated and analysed in different iterations of the same pipeline. PlasmoDB v48 annotations (supported by GeneDB) and genome assemblies were used [10, 33].

For the *P. berghei* datasets the liver stages, and some of the asexual blood stages (rings, trophozoites and schizonts), were defined by time of development in culture. In some published data sets, male and female gametocytes were analysed separately, while in others gametocytes were mixed. The 6 h Liver samples from [34] were removed due to low read counts, also removed by the authors in the original publication. Additionally, a 2 h Liver sample (SRR11142819) from [35], and two schizont samples (SRR3437888 and SRR3437912) from [36] were removed because hierarchical clustering showed that they were dissimilar to the other samples.

The gene counts were imported into R v4.0.2 where they were normalized by gene length and library size into TPM (R Core Team, 2018). TPM was chosen over RPKM/FPKM (RNA/Fragments Per Kilo) since TPM always totals 1 million in a given sample, while per sample sum of RPKM varies [37].

Pearson correlation (Additional file 7: Fig. S1A) and Principal Components Analysis (PCA; Additional file 8: Fig. S1B) demonstrate that samples from the same life cycle-stage(s) in different experiments robustly cluster together. PCA was calculated using R’s prcomp function and the variables were standardized and scaled ('center’ and ‘scale.’ set as TRUE). It was concluded that gene expression could be accurately compared between studies, and thus the datasets were combined together to obtain one average sample per life-cycle stage (Additional file 2). Mean expression was calculated, and the median was not used because this leads to the TPM of groups no longer adding up to a constant value. Some life cycle stages from slightly different times or conditions were grouped together as global gene expression was similar, such as liver stage samples at 4 h post-infection or earlier (Liver.pre4h), liver stage samples at 48 h post infection or later (Liver.post48h), the chronic and acute samples from Brugat et al. (Asexual.Mixed) [20], the different times of ookinete development from Otto et al. (Ookinete) [12], the ‘activation’ state of gametocytes from Ukegbu et al. (Gametocytes) [25], 3 h post-uptake and earlier blood meal samples from Ukegbu et al. (BloodMeal-Pre3h) [25], and the liver stages from different cell lines.

The annotations of *pir* genes in each species were taken from PlasmoDB v48 and can be found on the first sheet of Additional file 2 (*P. berghei*) and the second sheet of Additional file 6 (*P. chabaudi*). Note that this version of PlasmoDB annotated the orthologous genes PBANKA_0524600 and PCHAS_0524800 as members of the *pir* family, but this is likely to be erroneous due to their two-exon structure and lack of similarity with other *pir* genes. Hence, they were excluded from analysis here. The ChAPL/AAPL information for *P. chabaudi* was taken from Brugat et al. [20].

R packages used in manipulating and analysing the data include dplyr [38], data.table [39], reshape2 [40], stringr [41], tibble [42], readr [43], and readxl [44, 56]. Visualization was achieved using ggplot2 [45], ggpattern [46], RColorBrewer [47], corrplot [48], plotly, viridis [49], circlize [50], and ComplexHeatmaps [51].

### Deconvolution of *P. chabaudi* bulk transcriptomes

To determine the relative proportions of different life stages in the *P. chabaudi* transcriptomes, the approach described in Aunin et al. [52] was used. Briefly, pseudobulk samples (excluding mosquito and liver stages) derived from the Malaria Cell Atlas [14] were used as a reference to deconvolute with CIBERSORT v1.06 using default settings [53].

### Statistical analysis

Statistical analyses of gene expression were performed on R v4.0.2. For sets of genes (such as all *pir* genes, sub-families, and Short or Long-form groups) Qusage v.2.22.0 [54] was used with the TPM data, using 2e18 iterations. For individual genes DESeq2 v1.28.1 [55] was used, applying apeglm v1.10.0 [56] log fold change shrinkage to reduce the impact of lowly expressed genes on the differential expression analysis. Individual p values for comparisons were collated and multiple testing correction by the false discovery rate was calculated using R’s p.adjust function. In each case the different biological replicates from every experiment were treated as independent samples.

### Phylogeny

A ‘transformation by orthology’ search was conducted on PlasmoDB v48 using *P. berghei* ancestral gene *PBANKA_0100500,* which uses OrthoMCL for this purpose [57]. The *P. c. chabaudi* (CB strain) genome was not on PlasmoDB at the time of analysis so this ancestral gene was identified using a BLAST search with the AS strain gene. With nineteen orthologs from the different *Plasmodium* species, MUSCLE (v3.8.31) alignment [58] and PhyML (v3.1) tree-building with LG model 100 bootstrap replicates (otherwise default settings) [59] was conducted on SEAVIEW v4.7 [60]. The tree was imported to R and some additional strains of the same species were removed to simplify the final tree. The R packages used for tree-design were treeio v.1.12.0 [61], ggtree v2.2.1 [62] and ape v5.4 [63].

### Ancestral *pir* gene transcriptional analysis

The orthologs of the ancestral *pir* gene were then investigated to determine whether it is also highly transcribed in other *Plasmodium species* for which transcription data is available. TPM/RPKM from published studies (Table 1) was used to calculate the proportion of ancestral *pir* expression relative to the rest of the transcriptome.

**Table 1:** RNAseq experiments included in the analysis for Fig. 5

References	Species	Stage
[81]	*P. vivax*	Asexual.Schizont
[82]	*P. yoelii*	Gametocytes
[83]	*P. vinckei*	Asexual.Mixed
[77]	*P. c. chabaudi* CB	Asexual.Mixed
[84]	*P. ovale*	Asexual.Mixed
[85]	*P. cynomolgi*	Liver.Schizonts/Hypnozoites
[86]	*P. coatneyi*	Asexual.Mixed

### Data availability

The RNA-seq data relating to *P. c. chabaudi* (AS strain) intraerythrocytic developmental cycle are available from the ENA (ERP002273). The relationship between individual samples and ENA accessions is described in Additional file 16: Table S1.

## Results

### Asexual blood stages, liver merozoites and male gametocytes are the foci of *pir* gene expression in *Plasmodium berghei*

**Fig. 1:**
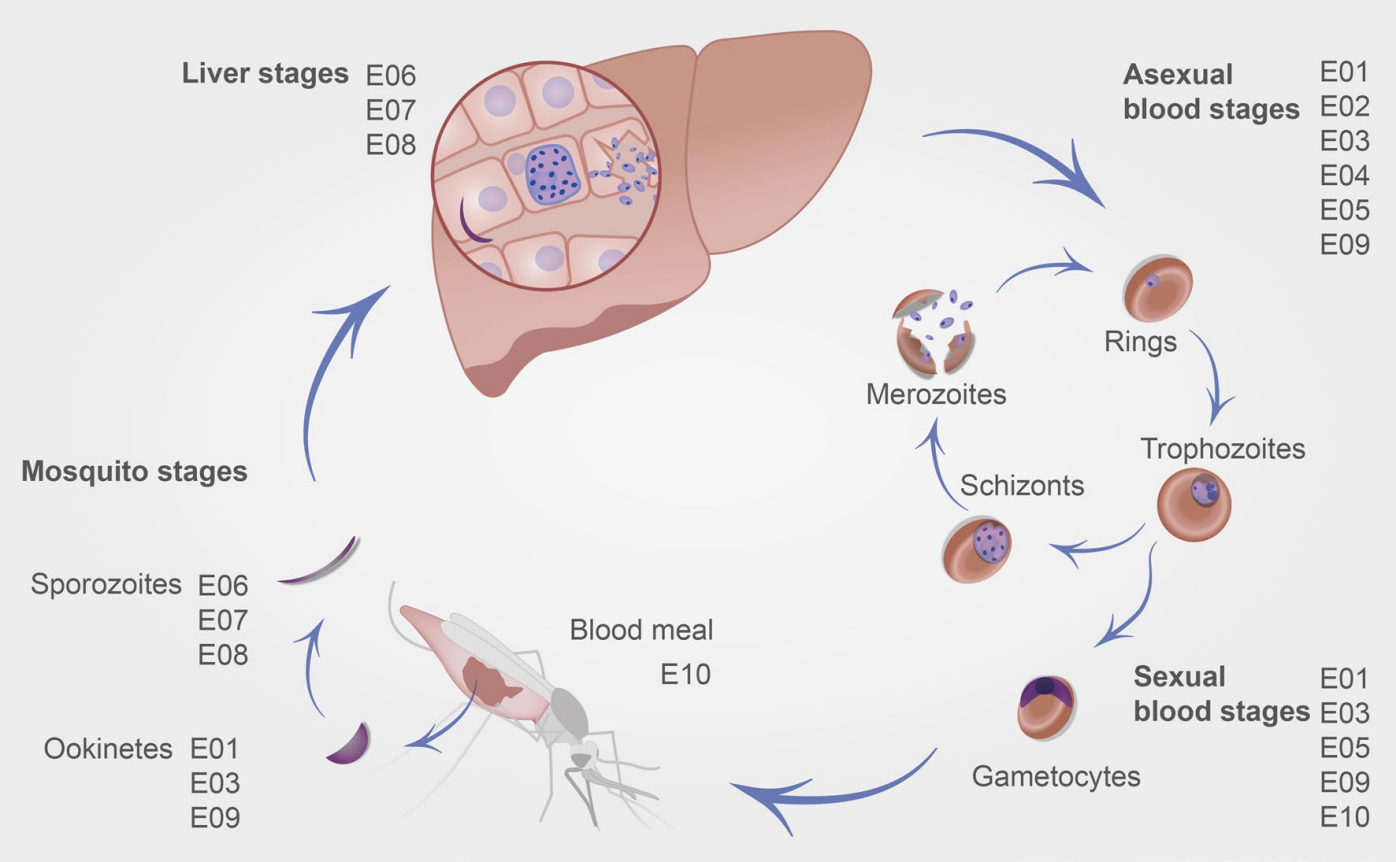
Systematic study of *P. berghei* RNAseq datasets that covers its entire life cycle and demonstrates the dynamic expression of *pir* genes throughout. Schematic of the published experiments used in this study and the *P. berghei* specific developmental stages during which samples were collected. The experiment codes (E01 etc.) correspond to the references in Table 2

The rodent *pir* gene family is divided into two groups named S and L based on sequence similarity and average gene length [12]. These are further classified into subfamilies (L1–L4; S1–S8), with some being unique to a particular rodent species (e.g. S7, *P. chabaudi*; S8, *P. berghei*). In order to determine whether an association exists between transcription of any *pir* subfamily and a particular life cycle stage, a systematic analysis of published transcriptome studies from the rodent *Plasmodium* species, *P. berghei* (ANKA strain) was performed (Fig. 1, Table 2, and Additional file 1). These included samples from most of the life cycle stages of the parasite in both the mosquito and mouse hosts.

**Table 2:** *P. berghei* ANKA datasets

Experiment code	References	Stages
E01	[12]	Asexual: Rings; Trophs; Schizont; Gametocytes; Ookinetes
E02	[20]	Asexual: Mixed
E03	[36]	Asexual: Schizont; Gametocytes; Ookinetes
E04	[78]	Asexual: Mixed
E05	[79]	Asexual: Mixed; Gametocyte: Female; Gametocyte: Male
E06	[34]	Sporozoites; Liver: 24 h; Liver: post 48 h (48 h, 54 h and 60 h); Liver: Merozoites
E07	[80]	Sporozoites; Liver: 24 h; Liver: post48h (48 h)
E08	[35]	Sporozoites; Liver: Pre4h (2 h and 4 h); 12 h; 18 h; 24 h; 36 h; 48 h
E09	[24]	Asexual: Mixed; Gametocyte: Female; Gametocyte: Male; Ookinetes
E10	[25]	Blood Meal: Pre3h (1 h and 3 h); Blood Meal: 24 h; Ookinetes

The gene expression values (TPM; transcript-per-million) for the different samples across all genes were used for data normalization. The individual samples from different experiments separated robustly by stage of development of the parasite (Additional file 7: Fig. S1A, Additional file 8: Fig. S1BC). As sexual stages may be present in host blood, the transcriptional activity of selected sexual stage markers was used to confirm the relative purity of asexual blood stage samples from across the multiple experiments (Additional file 9: Fig. S2). The genes selected were *p28* (*PBANKA_0514900*) [64] and *nek4* (*PBANKA_0616700)* (female gametocytes and ookinetes) [65], *mapk2* (*PBANKA_0933700*) (male gametocytes) [66], and *hap2* (*PBANKA_1212600*) (gametes) [67]. Their transcriptional activity confirmed that the asexual blood stages had few gametocyte contaminants.

**Fig. 2:**
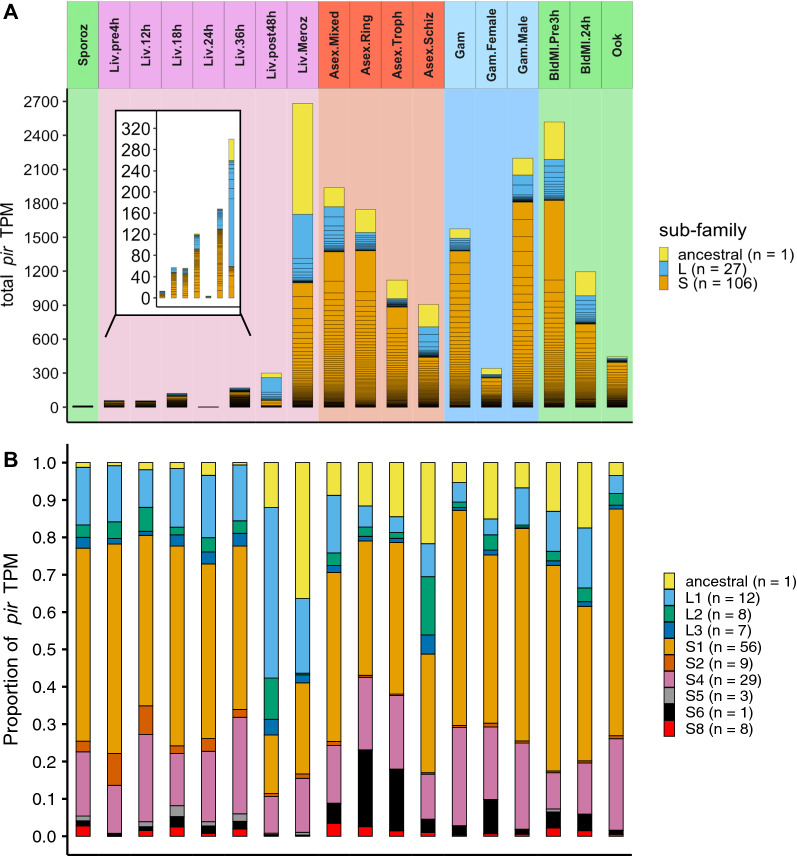
Expression of *pir* genes throughout *P. berghei* life cycle. **A** Bar chart of total *pir* gene TPM (y-axis), calculated from the mean expression across all experimental samples of each life cycle stage (x-axis). The colours of the bar chart denote the classification; ancestral (yellow), Long (L) (blue) and Short (S) (orange), with the boxes of the stacks denoting expression of the individual genes. The background and strip name colours correspond to groups of life cycle stages, including mosquito (green), liver (pink), asexual blood (red), and gametocyte (blue) stages. The genes in each subfamily are ordered by expression levels. Inset of figure shows an enlargement of the sporozoite and liver stages. **B** Bar chart of the proportion of total *pir* gene TPM (y-axis), contributed by the ancestral *pir* (yellow) and family sub-divisions of the Short/Long *pir*s, for each stage (x-axis). Stages are separated as in (**A**). Legend shows the number of genes that are members of each grouping, which are collated together for each box in the stacked bars. The ordering is by family sub-division, from ancestral, to Long groups (1–3) and finally Short groups (1–2, 4–6, 8)

The genome of *P. berghei* (ANKA strain) contains 134 *pir* genes. Although multiple *pir* genes are transcribed at all stages of the life cycle (Fig. 2A), this begins at very low levels in salivary gland sporozoites and early liver stages, and is then followed by an increase in both the level of transcript, and the total number of *pir* genes transcribed, in the later liver stages. One study analysed RNAseq data from liver merozoites [34], and showed that they display a dramatic increase in the levels of *pir* gene transcripts over earlier liver stages. High levels of transcript are maintained in asexual blood stages declining somewhat by the schizont stage. There are relatively few *pir* transcripts in female gametocytes, whereas the level of *pir* transcription in male gametocytes is of a similar or greater magnitude and breadth to that observed in asexual blood stages. Ookinetes within the mosquito midgut transcribe fewer *pirs* at lower transcription levels, similar to late liver stages, and female gametocytes.

There is one subfamily of *pir*s, distinct from S and L, containing only a single member. It has syntenic orthologues in the genomes of rodent and primate-infecting *Plasmodium* species. This *pir* and its orthologs have been described as the ancestral *pir*, because the other subfamilies may have derived from it [20, 21]. The *P. berghei* ancestral *pir* orthologue (PBANKA_0100500) is consistently highly transcribed from late liver stages, through asexual intraerythrocytic development and in blood stage gametocytes (shown in yellow in Fig. 2A, B), dominating the *pir* transcriptional profile whenever *pir*s are highly expressed.

The transcription of L- and S-*pirs* in sporozoite and early liver-stage parasites broadly reflects the genome composition (20% L, 79% S). However, in the later liver stages (post 48 h) L-*pirs*, predominantly of the L1 clade, comprise a distinctly higher proportion of the transcripts (61% of TPM; Fig. 2B). The increase in expression of the *pirs* between the 24 h liver stages and the post 48 h liver stages is concentrated in the L-forms (all *pirs* between liver post 48 h and 24 h: log2 fold change 0.67, FDR 0.029; L-form pirs: log2 fold change 1.56, FDR < 5 × 10^–9^).

Early intraerythrocytic asexual stages show a lower proportion of L-*pir* transcripts (7–9% in rings and trophozoites), but rising to 33–34% in schizonts—dominated by L2 *pirs*. The stage with the next highest proportion of L2 *pirs* is late liver stages (post 48 h), suggesting that the highest proportion of L2 transcripts is associated with development of extracellular invasive forms (merozoites). L1 *pirs* have highest proportions of total *pir* transcripts in the late liver stages. Note that it has previously shown that *P. berghei* L2s have more similar sequence properties to *P. chabaudi* L1s despite their names [20]. The gametocyte stages express slightly lower relative proportions of L-*pir* transcript (7–11%). Members of the S sub-family form a greater proportion of the *pir* transcriptome of rings, trophozoites and gametocytes (Fig. 2A, B), with the largest subfamily, S1 (Fig. 2B), making up the majority of *pir* transcripts.

Analysis of gene expression of the subfamilies in those samples which exhibit high levels of *pir* transcriptional activity (male gametocytes, liver merozoites and mixed asexual stages) (Additional file 3) showed differential expression of multiple sub-families. Each of these samples has a distinct *pir* transcriptome; however, no single sub-family is uniquely associated with a particular stage, as there is overlap between the *pirs* transcribed at each stage. Sub-families are up or downregulated in tandem with the rest of the *pir* family, and only a few enrichments can be found in given stages of the life cycle. However, there is an association between L-form *pir* and merozoite production.

Although transcription of individual *pir* genes tended not to be stage-specific, there were *pir*s clearly enriched in certain stages of the life-cycle (Additional file 4). These included the L1 *pir*s PBANKA_0317181 in male gametocytes (compared to mixed asexual stages: log fold change 5.09, FDR < 5 × 10^–9^; compared to liver merozoites: log fold change 10.65, FDR < 5 × 10^–7^) and PBANKA_0600031 in liver merozoites (compared to mixed asexual stages: log fold change 5.69, FDR < 5 × 10^–7^; compared to male gametocytes: log fold change 6.82, FDR < 5 × 10^–6^). The *pir* gene PBANKA_0400500 was highly transcribed in two of the three experiments performed for the later liver stages (post.48 h). This gene is not restricted to liver-stage expression, although notably high in the liver stages, suggesting that it may play a role in late exo-erythrocytic and early asexual stage parasites.

### Differential timing of expression of L and S *pir* gene subfamilies during the blood cycle of *P. chabaudi*

These data show that *pir* gene expression levels in *P. berghei* are low in mosquito stages, but high in the mouse from late liver to asexual blood stages and male gametocytes. To explore the expression of rodent *pir* genes at higher resolution across the asexual blood stages and to determine whether there were more subtle differences in expression of the different subfamilies transcriptional data from another rodent malaria parasite, *P. chabaudi chabaudi* AS (Additional file 5) was generated. This parasite has a largely overlapping repertoire of *pir* genes. In this model, *pir* gene expression at 14 h post infection is well understood and it can be transmitted by mosquito. Compared to serial blood passage mosquito transmission results in expression of a wide repertoire of *pir* genes which is likely to be more representative of the situation in the wild [20, 23].

**Fig. 3:**
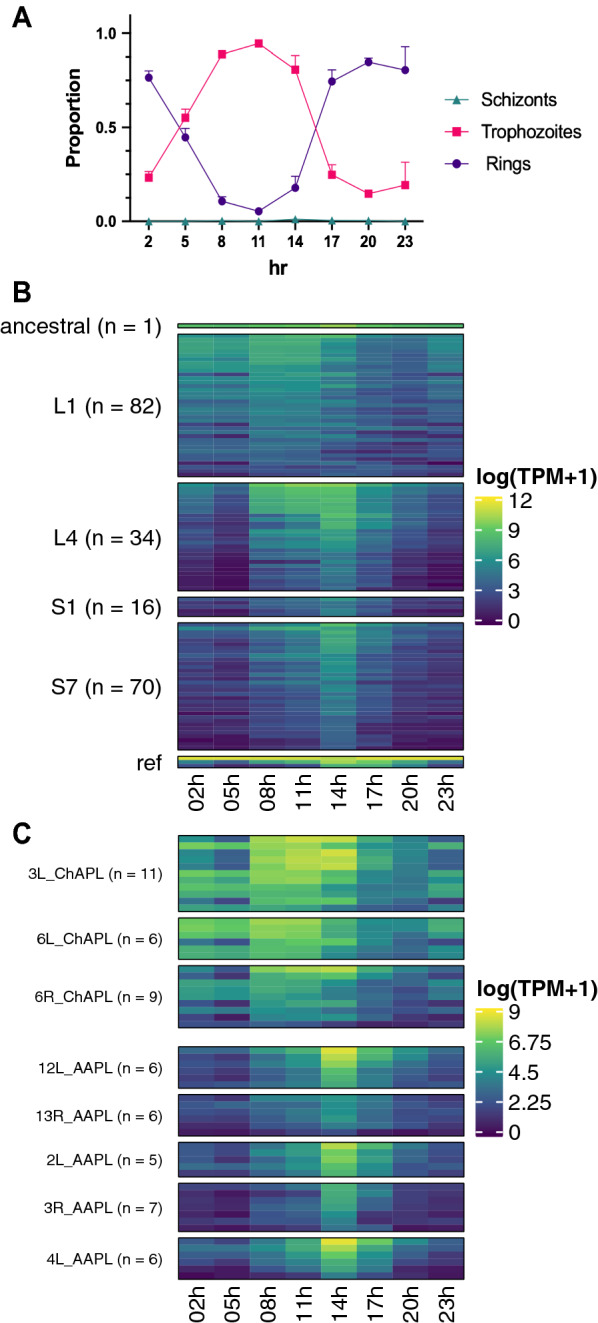
Intraerythrocytic development and *pir* gene expression across the 24 h asexual blood cycle of *P. c. chabaudi* AS parasites transmitted by RMT.** A** Microscopic counts of ring, trophozoite and schizont stage parasites in Giemsa-stained blood smears, as a proportion of the total parasitaemia (n = 3). Graphs show mean (± SD). Value 1 would indicate that all parasites counted, are of the specific developmental stage. **B** Heatmap of *pir* gene expression, log(TPM + 1), at each timepoint. Genes are grouped according to sub-family (L1, L4, S1 and S7) and ordered by mean expression across the timepoints within each sub-family, with the ancestral *pir* at the very top*.* Three blood stage-specific genes were included for comparison ‘ref’: MSP1 (highly transcribed in schizonts/merozoites), HSP70 (highly transcribed in rings/trophozoites) and AMA1 (highly transcribed in schizonts/merozoites). Genes expressed under 1 TPM in every sample were removed. **C** Heatmap of transcription of the *pir* genes assigned to ChAPL or AAPL loci [20], log(TPM + 1), at each timepoint, grouped according to locus

Microscopic analysis of the proportions of ring and trophozoite forms at each time point shows that development is largely synchronous (Fig. 3A; Additional file 10: Fig. S3; Additional file 6). A deconvolution approach using previously published single-cell RNA-seq data [14] confirmed the enrichment of the individual stages at the appropriate time points (Additional file 11: Fig. S4A). Principal Components Analysis of the RNAseq time-points confirmed that most of the different samples form a continuum of transcription over time, with a ‘gap’ between 14 h/17 h around when schizonts develop and sequester. This verifies the robust capture of the trophozoite and ring stages of the 24 h asexual blood cycle (Additional file 12: Fig. S4B).

*Pir* genes show a cyclical expression pattern across the developmental cycle, with the majority upregulated at 8–14 h (trophozoite stage) (Fig. 3B; Additional file 6). This signal suggests that *pir* genes may be required in the schizonts and merozoites, as the transcriptional signal is expected to precede translation of protein by several hours [68]. Indeed, antibodies generated to specific PIR proteins showed maximal reactivity to parasites at trophozoite and schizont stages; with fewer *cirs* being detected at ring stages and no reactivity to early ring stage, leading these authors to hypothesize that the protein is stable during parasite maturation but with little transfer from merozoite to newly invaded red blood cells [19]. *Pirs* upregulated at ring stages were predominantly of the L1 subfamily, which have a slightly earlier transcriptional peak (8–11 h) in the developing trophozoites and are quiescent only in the early ring stages. Transcription of both the S7 and S1 subfamilies peaks sharply at 14 h. L4s notably exhibit two distinct temporal *pir* transcription profiles, with a proportion being similar to L1s and the remainder being like the short S *pirs* where (Fig. 3B). The ancestral *pir* gene, PCHAS_0101200, is transcribed throughout the intraerythrocytic developmental cycle, but still peaks during the trophozoite stages (Fig. 3B and Additional file 13: Fig. S5), as observed above for *P. berghei*. This gene is one of the most highly transcribed *pir* genes, second only to the *pir* PCHAS_0600600.

Brugat et al. [20] described distinctive clusters of *pir* genes associated with the acute (Acute-Associated *pir* loci or AAPL) and chronic (Chronic-Associated *pir* loci or ChAPL) phases of Mosquito Transmitted (MT) *P. chabaudi* infection. Here, temporal transcription of clusters of *pirs* at ChAPL loci on chromosomes 3 and 6 mirrors that of L1s, and early peaking L4s, as expected, as most ChAPL *pirs* are L1s, while clusters at AAPL loci, mostly S7s and late peaking L4s, peak sharply in late trophozoites (14 h) (Fig. 3C), suggesting that they perform separate functions during parasite development.

### The putative ancestral *pir* gene is a distinctive target for functional studies

**Fig. 4:**
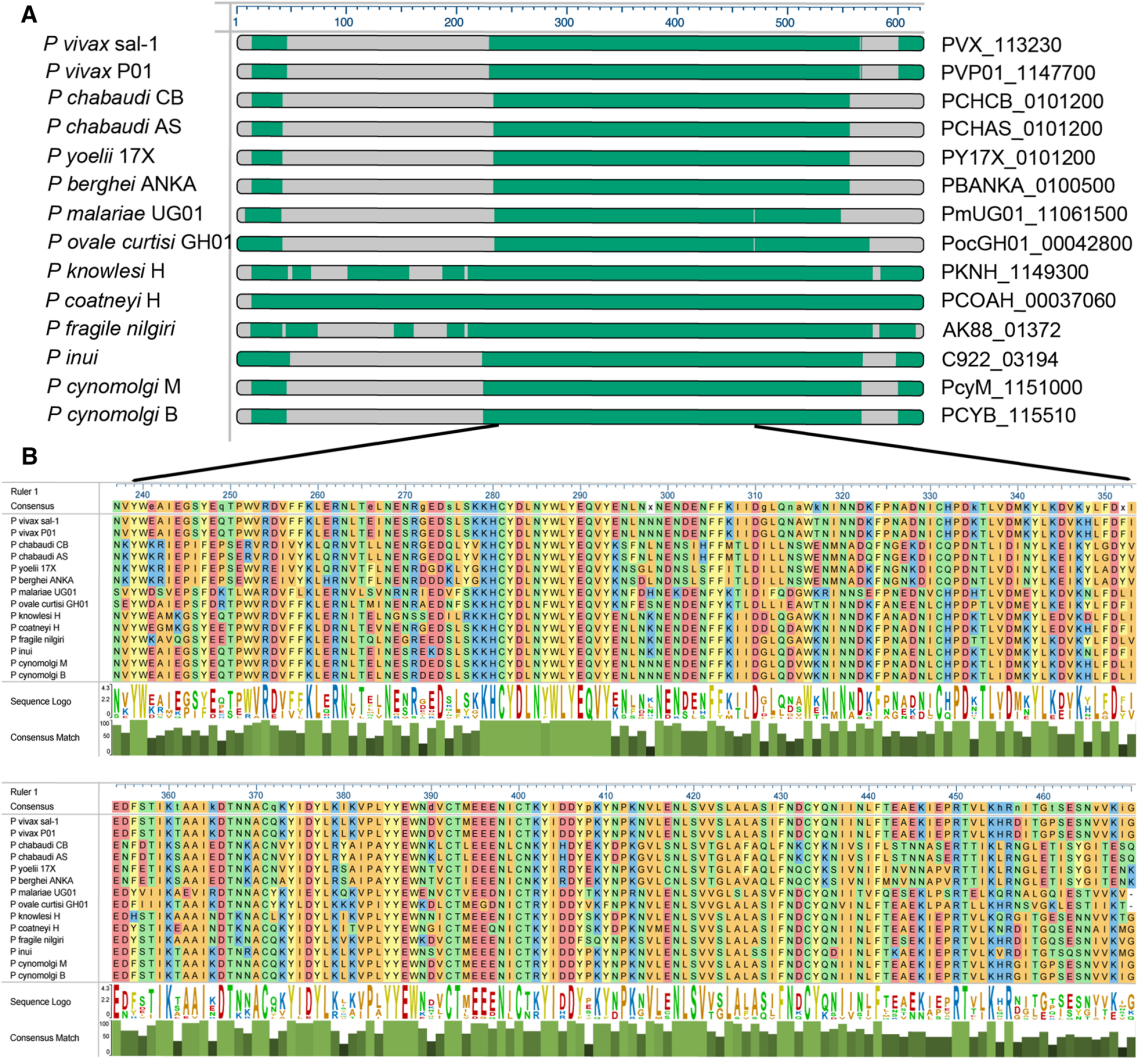
Conservation and gene expression of the ancestral *pir* across the *Plasmodium* genus. **A** Sequence alignment of 15 ancestral *pir* orthologs (i.d.verified 20210527) identified in different *Plasmodium* spp. indicating regions and sequence of high conservation. **B** Expanded region of the most highly conserved section of 234 aa, illustrated with a sequence logo and percentage match of the consensus

**Fig. 5:**
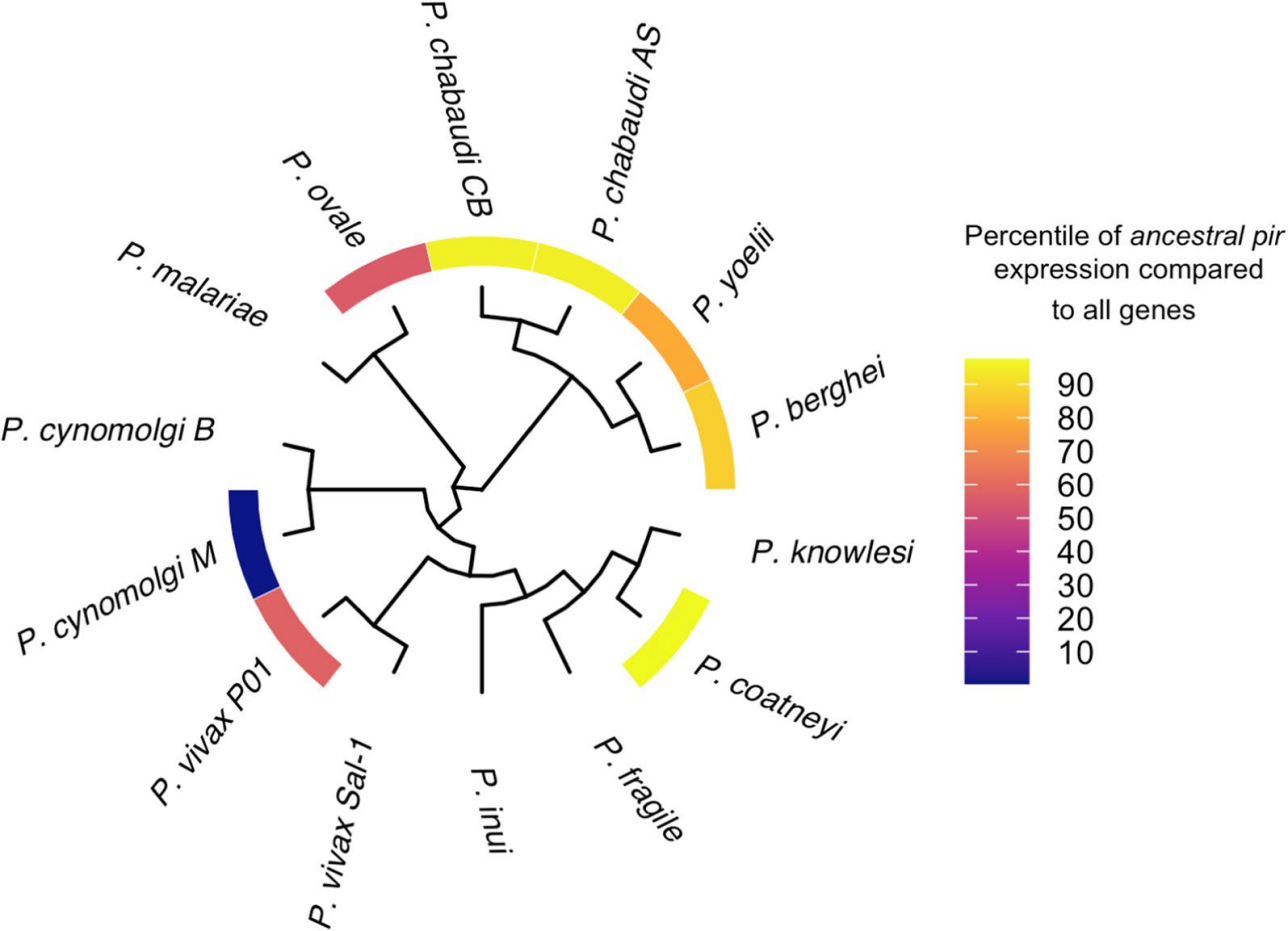
Phylogenetic analysis of the ancestral *pir* gene. Cladogram of the 15 orthologs, with heatmap of expression of the gene relative to the rest of the genome, where data is available (from multiple transcriptomic datasets as RPKM or TPM—see Table 1). With the exception of *P. cynomolgi* strain M the ancestral ortholog is expressed within at least the top 50% of genes and usually in the top 30% of genes

The *P. berghei* ancestral *pir* orthologue, *PBANKA_0100500,* is the single most highly transcribed *pir* gene in this species, shown above. It contributes a high proportion of the total *pir* steady-state transcriptome in late liver, asexual blood and gametocyte stages. Transcription is only diminished in stages where overall transcription of this multigene family is low i.e. liver stages before 36 h of development, ookinetes and sporozoite stages. Similarly, in *P. chabaudi* AS, the ancestral *pir* orthologue (PCHAS_0101200) is highly transcribed across the complete asexual developmental cycle (Additional file 13: Fig. S5). Extending earlier findings a single, syntenic ortholog was found in all species whose genomes contain canonical *pir*s [10, 20, 21] (Fig. 4A). The sequences are highly conserved between species, with multiple blocks of 90–100% similarity (Fig. 4B). The gene is within the top 50% of expressed genes in all species for which RNAseq data could be found (Fig. 5).

## Discussion

It has been shown above that high numbers of *pir* gene transcripts were expressed from a variety of family members in stages which produce the invasive blood forms (merozoites) and in male gametocytes. Conversely, only low levels were expressed throughout the mosquito stages, early vertebrate stages and in female gametocytes. This strongly indicates that this multigene family is not required for development within the mosquito beyond sexual reproduction. Instead, it is likely that the bulk of this large family, at least in rodent malaria parasites, is involved in blood stages, leading from red cell invasion by first generation (liver) merozoites to fertilization and generation of the ookinete form rapidly after mosquito blood feeding. The high levels of *pir* gene expression in *P. chabaudi* parasites in asexual blood stages [20, 22, 23] and the specificity of *pir* gene expression in male *P. berghei* gametocytes versus female gametocytes [13] have previously been shown. This new analysis looks broadly and deeply, confirming the findings based on much lower coverage Malaria Cell Atlas data [14].

This study shows that L *pir* genes are highly expressed in late liver stages, merozoites and schizonts. These would require some time before being translated into functional proteins and it may be that they function in rings, e.g. soon after red cell invasion. Expression of S-type *pirs* is more steady throughout the development cycle. L-type *pir*s are first transcribed in mature liver schizonts, and upregulated again in asexual blood schizonts, so they are potentially involved in merozoite formation/function. When investigated in more detail at this part of the lifecycle in *P. chabaudi* dynamic expression of each individual *pir* gene occurs, as has been shown for genes across the *Plasmodium* genome [69]. Again, L and S types behave differently, suggesting differential regulation and function. Underlying this pattern is the relatively early and broad expression of the ChAPL loci, which are rich in L1 and a subset of L4 pirs, so they may be required throughout the cycle. The S-rich AAPLs peak later and more sharply, and may only be needed for one stage of the asexual cycle. ChAPLs have previously been shown to be associated with chronic infections and it has been postulated that populations of parasites expressing ChAPLs survive the acute immune response, while those expressing AAPL loci are killed [20]. Whether this relates to a function in sequestration, evasion of adaptive immunity or a completely different function is unclear. Molecular mimicry as a means of immune suppression has been described for *P. falciparum* RIFINs, another sequence-variable multigene family, some of which mimic the natural ligand for LILRB1 [70] and suppress the activity of NK cells. *Plasmodium knowlesi pir* sequences have also been shown to have a striking resemblance to 50% of CD99, a T-cell regulating protein, suggesting molecular mimicry and potential immune-modulating activity [8]. Such interactions could promote chronicity, although the mechanisms of the contribution of AAPLs and ChAPLs in infection outcome have yet to be elucidated.

The other focus of *pir* gene expression is in male gametocytes, with a relative absence of expression in females. A small number of L1 *pir* genes are highly expressed at this stage in *P. berghei*, but S1s and S4s predominate. Could this represent a continuation of function from asexual to sexual parasites? Male gametocytes are found in the bloodstream, whereas immature female gametocytes reside in bone marrow, primarily in the extravascular space. Here, cellular rigidity may be more important than the receptor-mediated interactions of sequestration within the vasculature [71–73].

These analyses indicate that expression of multiple *pirs* occurs in the parasite developmental stages which are predominantly circulating in the blood, where the parasite-infected cells are targeted by the host’s immune system. PIR proteins have been demonstrated to be targeted by antibodies [6] and several studies have shown localization at or near the parasite surface [16, 19, 74]. Recent structural studies together with sequence analysis and modelling have revealed hydrophobic conserved disulphide bonds forming cysteine residues suggesting that part of the PIR protein may be extracellular [34, 75]. It is hypothesized that hydrophobic domains are exposed on the surface via a flexible linker. The diversity in sequence and length of the flexible loops are consistent with a surface location, and thus could interact directly with the host immune system or be involved in sequestration.

PIRs localize to different cellular compartments depending on the particular stage of asexual development e.g. RBC cytoplasm and parasitophorous vacuole [19, 76]. This raises the possibility that PIR proteins are likely to have functions in addition to immune evasion or immune-suppression in the mammalian blood stream. These transcriptional analyses do not shed light on this. However, an association between higher *pir* transcription and the stages involved in or just preceding proliferative steps of development, such as liver schizonts, asexual blood stages (specifically the trophozoites in *P. c. chabaudi*) and male gametocytes, was seen which could be indicative of a role in phase transition rather than overall heightened transcription in these stages.

Although the other major proliferation stage of the parasite, the oocyst in the mosquito, is not covered in the *P. berghei* bulk RNAseq, single cell RNAseq data suggest that *pir* transcription is low in more mature oocysts. However, the earlier oocysts may be high transcribers. Bulk RNAseq analysis of oocysts from rodent *Plasmodium* species at different times post-ookinete differentiation would provide crucial data to investigate this link.

The high level of transcription of one, conserved *pir* gene*,* across most *Plasmodium* parasites suggests that it may serve an important function in the parasite, again in the blood cycle. Transcription is upregulated just prior to entry into the blood stream and is maintained throughout the erythrocytic developmental stages. The *Plasmodium cynomolgi* RNAseq data demonstrated that transcription of its ortholog was much lower than for most of the transcriptome, however this data is from liver stages in which *pir* transcription may be low. Unlike most of the other *pir* family members vector transmission has little impact on the transcription levels of this gene [23, 77]. Although its distinctiveness suggests that it may fulfil a different role from other *pir*s, this gene may prove a more tractable target for future studies. It is anticipated that it will provide insights into the molecular function of the whole family.

## Conclusion

The *pir* gene family is the largest found in malaria parasites, with potentially important roles in virulence and chronic infection. The landscape of *pir* gene expression across the *P. berghei* life cycle has been characterized, highlighting the blood stages as the focus of activity. In depth analysis of the blood stages using the close relative *P. chabaudi* highlighted subtle differences in the timing of expression of different *pir* gene subfamilies. However, the most distinctive expression pattern found was for the putative ancestral *pir* gene, conserved across much of the *Plasmodium* genus, and very widely and highly expressed. The distinctiveness of this *pir* gene may make discovering its function more tractable while still shedding light on those genes already considered to be involved in host-parasite interactions.

## Supplementary Information

**Additional file 1.** Re-analysed counts of the *P. berghei* data from the studies listed in Table 2.

**Additional file 2.** *P. berghei* pirgene information, TPM values for individual samples and combined across life cycle stages, and summed across sub-families, chromosomes and loci.

**Additional file 3.** Statistics of *P. berghei* pirgene set comparisons (QUSage).

**Additional file 4.** Statistics of *P. berghei* individual pir genes (DESeq).

**Additional file 5.** *P. c. chabaudi* counts across the 24 hour asexual developmental cycle.

**Additional file 6.** *P.c. chabaudi* parasitaemia, pir gene information, TPM values, summed across loci, and z-scores.

**Additional file 7: Fig. S1.** (A) Pearson correlation plot of each transcriptome calculated from genome TPM values (0.04–1.00) for each stage from each experiment. The samples were clustered by hierarchical clustering using Ward’s clustering criterion (‘ward.d2’ setting in corrplot function [87], and six clusters are highlighted in boxes. Six clusters were chosen as most optimal with the ‘elbow method’ [88], as implemented through the factoextra package function ‘fviz_nbclust’ using k-means clustering and within cluster sums of squares. The Experiment codes are listed in Table 2.

**Additional file 8: Fig. S1.** (B) PCA plot of the genome TPM data coloured by the different stages and shapes determined by the originating experiment. PCA was conducted using the prcomp function in R. (C) PCA plot of the different samples by stage and experiment but using only *pir* gene TPM data, instead of the entire genome as used in (B).

**Additional file 9: Fig. S2.** Transcription of four gametocyte-specific marker genes across the life cycle stages of *P. berghei* in the different experiments. The genes included are *p28* (*PBANKA_0514900*) and *nek4* (*PBANKA_0616700*), markers for female gametocytes and ookinetes; *mapk2* (*PBANKA_0933700*), marker for male gametocytes [66]; *hap2* (*PBANKA_1212600*), gamete fusion protein [67]. Bar height corresponds to median TPM, with error bars showing the range of TPM values across replicates. Colours correspond to different experiments.

**Additional file 10: Fig. S3.** Representative Giemsa stained smears of *P chabaudi* infected iRBCs throughout the 24 h developmental cycle. Scale bar indicates 10 µm.

**Additional file 11: Fig. S4. **A Transcriptional deconvolution of each sample from every time point using scRNAseq data [14].

**Additional file 12: Fig. S4. **B PCA plot of the genome TPM data coloured by the different timepoints. PCA was conducted using the prcomp function in R.

**Additional file 13: Fig. S5.** Bar chart of the transcription of the ancestral *pir* gene (PCHAS_0101200) across the *P. c. chabaudi* AS asexual blood cycle. Each point represents one replicate and bars show the median TPM.

**Additional file 14. ** Table of contents of additional files 1–6.

**Additional file 15. ** Complete sequence alignment of the pirorthologues shown in Fig. 5A.

**Additional file 16: Table S1.** The relationship between *P. chabaudi* RNA-seq samples used in this study and their entries in the ENA is described.

## Data Availability

The RNA-seq data relating to *P. c. chabaudi* AS intraerythrocytic developmental cycle are available from the ENA (ERP002273). The relationship between individual samples and ENA accessions is described in Additional file 16: Table S1.

## Abbreviations

AAPL: Acute-associated *pir* loci
ChAPL: Chronic-associated *pir* loci
iRBC: Infected red blood cell
*pir/*PIR: *Plasmodium* Interspersed repeat
PCA: Principle components analysis
MT: Mosquito transmitted
RMT: Recently mosquito transmitted
