# Supplementary figures and images for: Analysis of pir gene expression across the Plasmodium life cycle
Source: Malar J. 2021 Nov 25;20:445. doi: 10.1186/s12936-021-03979-6 (PMC8614022; doi:10.1186/s12936-021-03979-6)

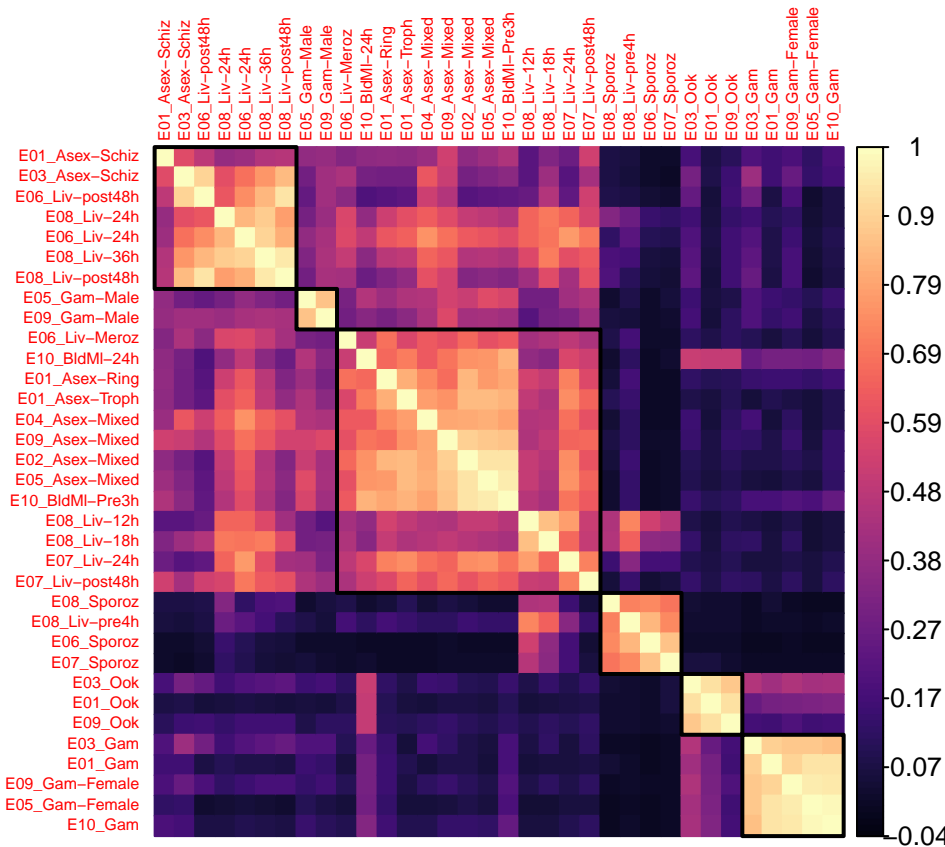

Supplement: Supplementary file 7 — Additional file 7: Fig. S1. (A) Pearson correlation plot of each transcriptome calculated from genome TPM values (0.04–1.00) for each stage from each experiment. The samples were clustered by hierarchical clustering using Ward’s clustering criterion (‘ward.d2’ setting in corrplot function [87], and six clusters are highlighted in boxes. Six clusters were chosen as most optimal with the ‘elbow method’ [88], as implemented through the factoextra package function ‘fviz_nbclust’ using k-means clustering and within cluster sums of squares. The Experiment codes are listed in Table 2. [file 12936_2021_3979_MOESM7_ESM.pdf]

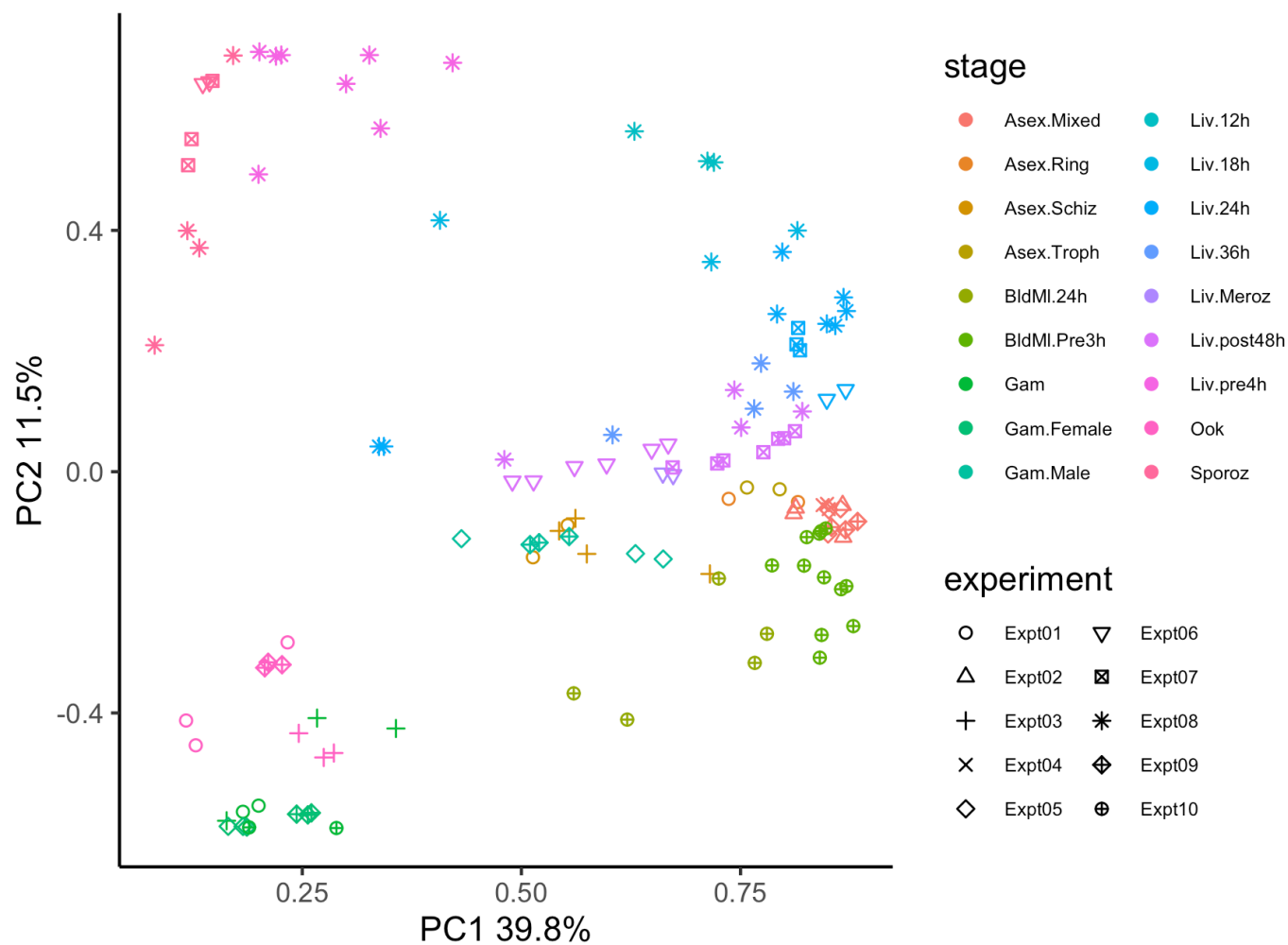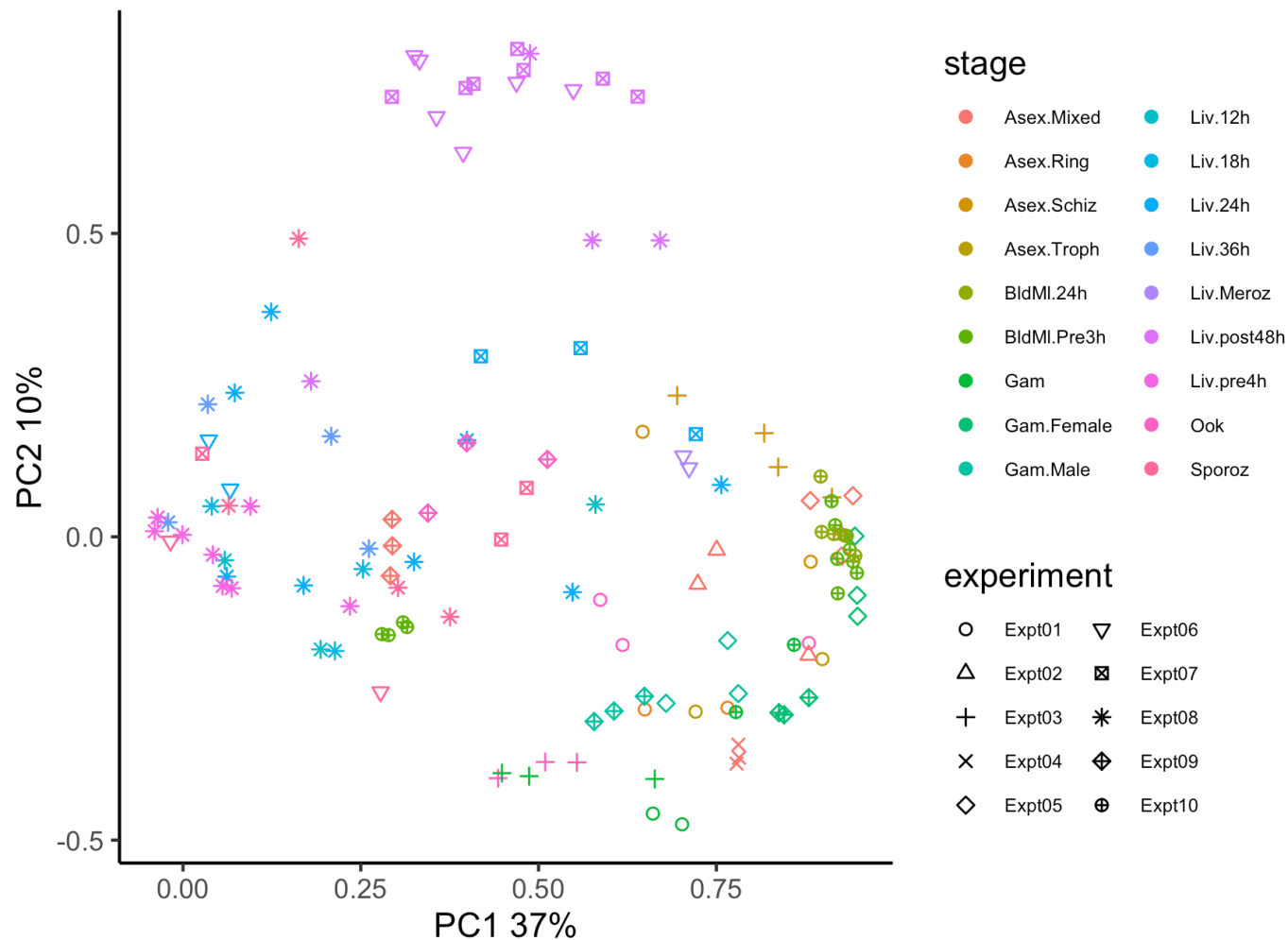

Supplementary Figure 1B/C

Supplement: Supplementary file 8 — Additional file 8: Fig. S1. (B) PCA plot of the genome TPM data coloured by the different stages and shapes determined by the originating experiment. PCA was conducted using the prcomp function in R. (C) PCA plot of the different samples by stage and experiment but using only pir gene TPM data, instead of the entire genome as used in (B). [file 12936_2021_3979_MOESM8_ESM.pdf]

total *pir* TPM

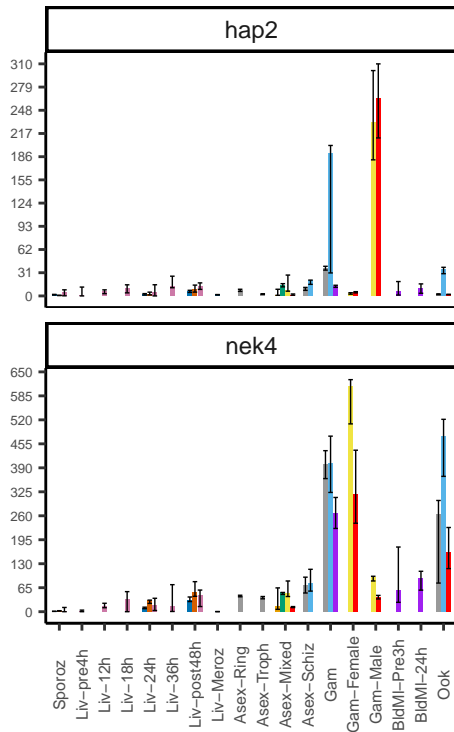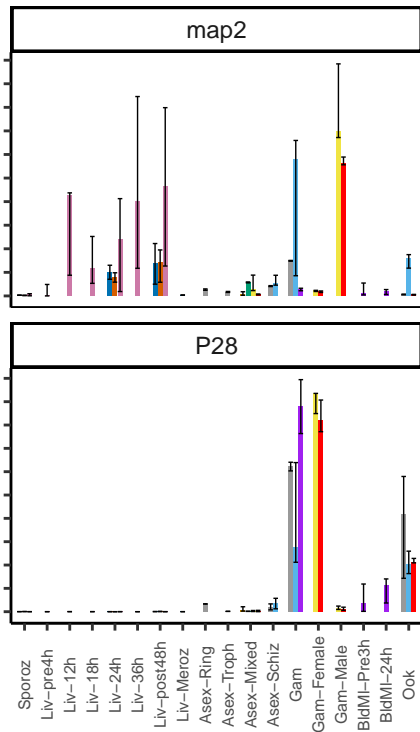

experiment

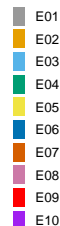

Supplement: Supplementary file 9 — Additional file 9: Fig. S2. Transcription of four gametocyte-specific marker genes across the life cycle stages of P. berghei in the different experiments. The genes included are p28 (PBANKA_0514900) and nek4 (PBANKA_0616700), markers for female gametocytes and ookinetes; mapk2 (PBANKA_0933700), marker for male gametocytes [66]; hap2 (PBANKA_1212600), gamete fusion protein [67]. Bar height corresponds to median TPM, with error bars showing the range of TPM values across replicates. Colours correspond to different experiments. [file 12936_2021_3979_MOESM9_ESM.pdf]

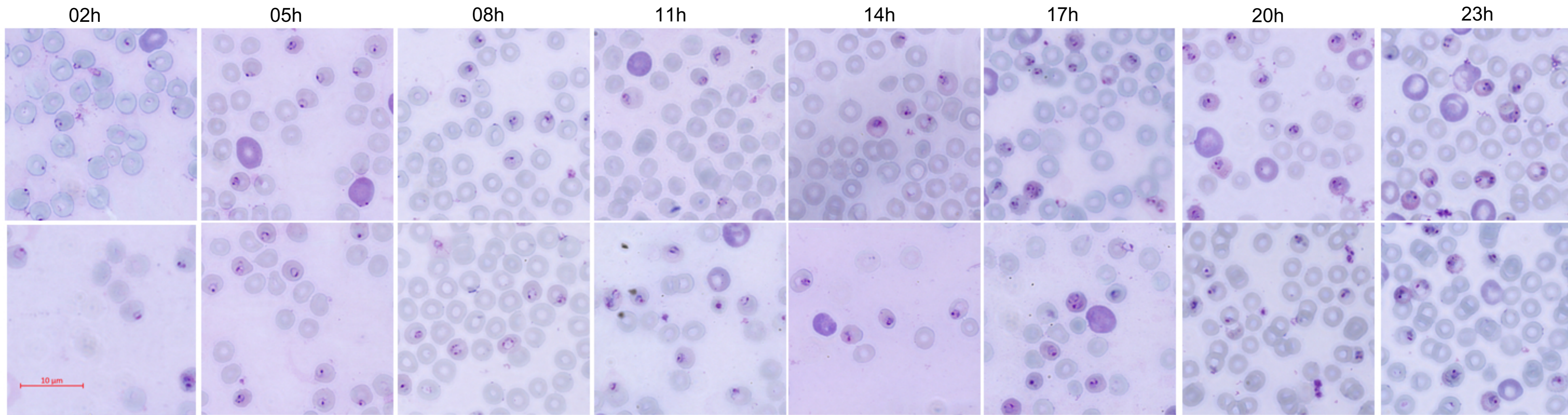

Supplementary Figure 3

Supplement: Supplementary file 10 — Additional file 10: Fig. S3. Representative Giemsa stained smears of P chabaudi infected iRBCs throughout the 24 h developmental cycle. Scale bar indicates 10 µm. [file 12936_2021_3979_MOESM10_ESM.pdf]

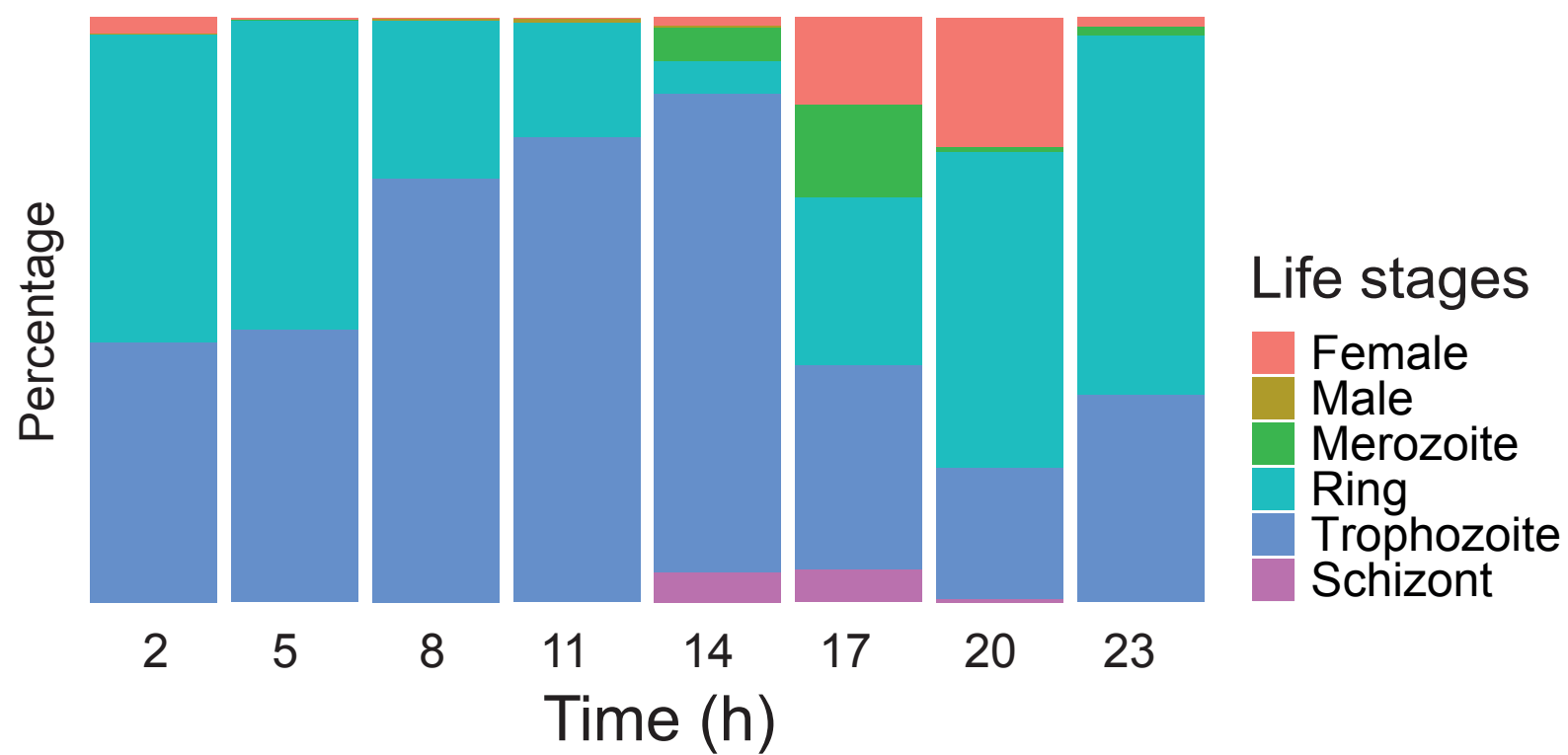

Supplement: Supplementary file 11 — Additional file 11: Fig. S4. A Transcriptional deconvolution of each sample from every time point using scRNAseq data [14]. [file 12936_2021_3979_MOESM11_ESM.pdf]

Supplementary figure 4

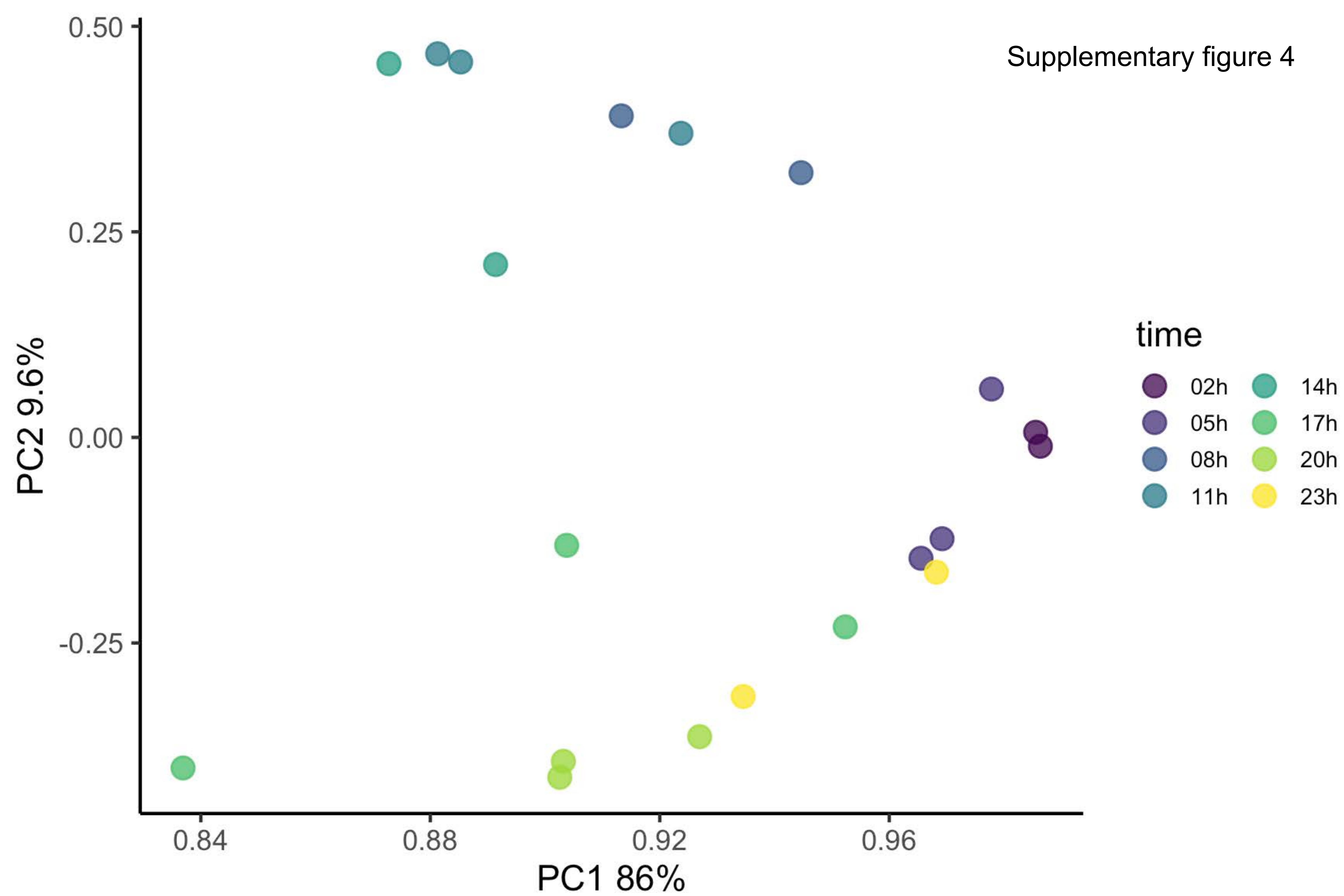

Supplement: Supplementary file 12 — Additional file 12: Fig. S4. B PCA plot of the genome TPM data coloured by the different timepoints. PCA was conducted using the prcomp function in R. [file 12936_2021_3979_MOESM12_ESM.pdf]

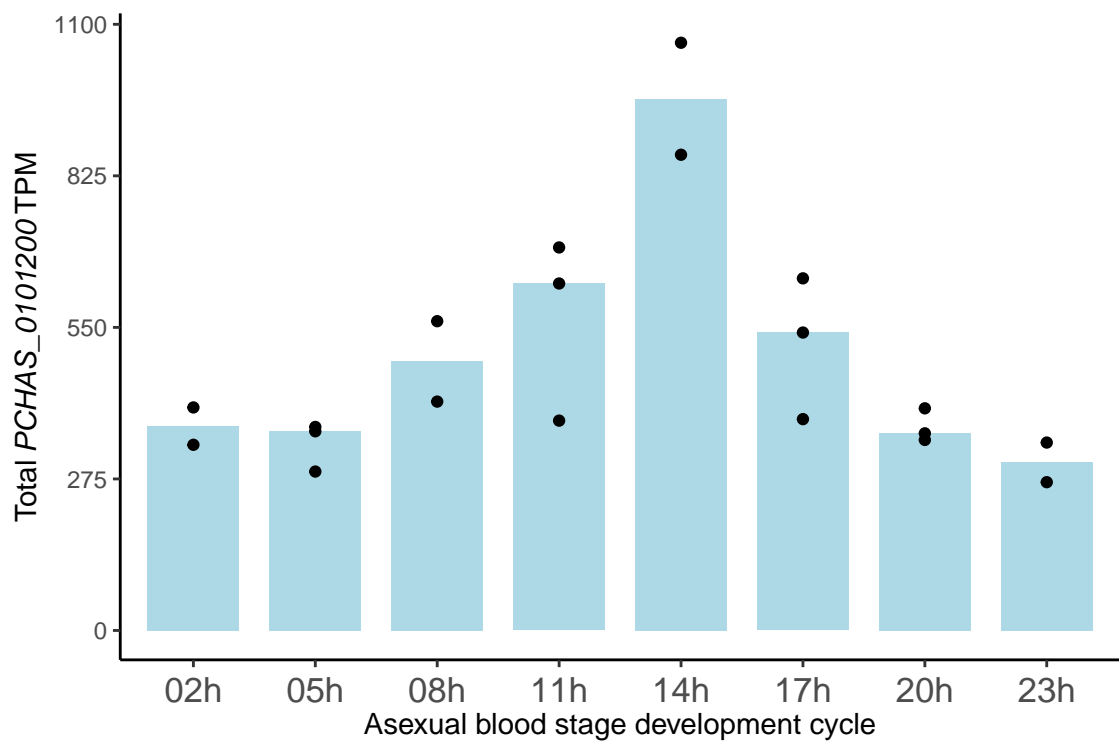

Supplement: Supplementary file 13 — Additional file 13: Fig. S5. Bar chart of the transcription of the ancestral pir gene (PCHAS_0101200) across the P. c. chabaudi AS asexual blood cycle. Each point represents one replicate and bars show the median TPM. [file 12936_2021_3979_MOESM13_ESM.pdf]
