# Supplementary material for: Analysis of pir gene expression across the Plasmodium life cycle
Source: Malar J. 2021 Nov 25;20:445. doi: 10.1186/s12936-021-03979-6 (PMC8614022; doi:10.1186/s12936-021-03979-6)
Supplement: Supplementary file 15 — Additional file 15. Complete sequence alignment of the pirorthologues shown in Fig. 5A. [file 12936_2021_3979_MOESM15_ESM.pdf]

<html><head></head><body><pre style="word-wrap: break-word; white-space: pre-wrap;">CLUSTAL O(1.2.4) multiple sequence alignment

```
PCHAS        --------------MEEEEKYEYLPAYEFYSKLNENVAEE--------------------       26
PCHCB        --------------MEEEEKYEYLPAYEFYSKLNENVAEE--------------------       26
PBANKA       --------------MEEEERYEHLPSYEFYNKLNENVSDE--------------------       26
PY17X        --------------MEEEERYEHLPSYEFYNKLNENVPDE--------------------       26
PKNH         --------------MKKDPKYKDLPSQIYYDKLNEDIVQDDEENEEP----EVNEDHGEN       42
AK88         -------------MGCYDPRYKDLPSQVYYAKLNEDIVEEEDD---GNEEHVENEEYEVN       44
PCOAH        --------------MKKDPKYKDLPSQKYYDKLNEDIVEEEDEEHEGNEEHEEHDEYGEN       46
C922         -MGVHRASTPNSMHWAKDSKYKDLPSQIYYDNLNEDIIEEEDDEDD--------------       45
PCYB         --------------MEKDPKYKDLPSYIYYDKLNEDIIEEDDE-----------------       29
PcyM         --------------MEKDPKYKDLPSYIYYDKLNEDIIEEDDE-----------------       29
PVP01        --------------MEKDPKYKDLPSQIYYDKLNEDIIEEEDD-----------------       29
PVX          --------------MEKDPKYKDLPSQIYYDKLNEDIIEEEDD-----------------       29
PVL          --------------MEKDPKYKDLPSQIYYDKLNEDIIEEEDD-----------------       29
PocGH01      MLQCGKLIHIYLFSIPQDPKYEHLPSYIFYEQLNSDINEDD-------------------       41
PmUG01       --------MNNGFYKLKDNTYNHLPSYKFYEQLNSDINEDD-------------------       33
                              :  *: **:  :* :**.:: ::

PCHAS        ------------------------------------------------------------       26
PCHCB        ------------------------------------------------------------       26
PBANKA       ------------------------------------------------------------       26
PY17X        ------------------------------------------------------------       26
PKNH         EEHEIND------ELEGNEENE---------------------------EHDEYGENEEH       69
AK88         EEQEVNEEEEV-------------------------------------------------       55
PCOAH        EENKVNEGNEENVEHEGNEGNVEHEENEGNVEHEENEGNVEHEENEGNELNEEYEENELN       106
C922         ------------------------------------------------------------       45
PCYB         ------------------------------------------------------------       29
PcyM         ------------------------------------------------------------       29
PVP01        ------------------------------------------------------------       29
PVX          ------------------------------------------------------------       29
PVL          ------------------------------------------------------------       29
PocGH01      ------------------------------------------------------------       41
PmUG01       ------------------------------------------------------------       33


PCHAS        ------------------------------------------------------------       26
PCHCB        ------------------------------------------------------------       26
PBANKA       ------------------------------------------------------------       26
PY17X        ------------------------------------------------------------       26
PKNH         DEYEENEGNEENEGNEEHEENEEHEENEEHDE------------------YEENEGN---       108
AK88         --------------------IEEQEVNEEEEVIEEQEVNEVQE-----------------       78
PCOAH        EEYEENEVNEENEGNEGSEEYEENEVNEENEVNEENEVNEENEGSEENEVNEENEGSEEN       166
C922         ------------------------------------------------------------       45
PCYB         ------------------------------------------------------------       29
PcyM         ------------------------------------------------------------       29
PVP01        ------------------------------------------------------------       29
PVX          ------------------------------------------------------------       29
PVL          ------------------------------------------------------------       29
PocGH01      ------------------------------------------------------------       41
PmUG01       ------------------------------------------------------------       33


PCHAS        ---------------------------------------------------DKEKPNKYW       35
PCHCB        ---------------------------------------------------DKEKPNKYW       35
PBANKA       ---------------------------------------------------DKEKPNKYW       35
PY17X        ---------------------------------------------------DKEKPNKYW       35
PKNH         ---------EEHDEYEENEGNEEHDEYEE---NEEYAEHGRNDELEVNKGNKGSAKNVYW       156
AK88         -------------VNEEQEVNEEQEVNEVQEVN---KVEQVNKEHGVNGQSKGIEENVYW       122
PCOAH        EVNEENEGSEENEVNEENEGSEENEVNEENEVNEGNEGNEQHEVNEVNEGSKGSEKNVYW       226
C922         ----------------------------------------ENDGNDENEESEESEENVYW       65
PCYB         -----------------------------------------NEETVENEESEESEENVYW       48
```

```
PcyM         -----------------------------------------NEETVENEESEESEENVYW       48
PVP01        -----------------------------------------E-----HEENEESQENVYW       43
PVX          -----------------------------------------E-----HEENEESQENVYW       43
PVL          -----------------------------------------E-----HEENEESQENVYW       43
PocGH01      -----------------------------------------------------EEVSEYW       48
PmUG01       -----------------------------------------------------NE-SVYW       39
                                                                     . **

PCHAS        KRIEPIFEPSERVRDIVYKLQRNVTLLNENRGEDQLYVKHCYDLNYWLYEQVYKSFNLNE       95
PCHCB        KRIEPIFEPSERVRDIVYKLQRNVTLLNENRGEDQLYVKHCYDLNYWLYEQVYKSFNLNE       95
PBANKA       KRIEPIFEPSEWVREIVYKLHRNVTFLNENRDDDKLYGKHCYDLNYWLYEQVYKNSDLND       95
PY17X        KRIEPIFEPSEWVREIVYKLQRNVTFLNENRDGDKLYGKHCYDLNYWLYEQVYKNSGLND       95
PKNH         EAMKGSYEQTPWVRDVFFKLERNITELNGNSSEDILRKKHCYDLNYWLYEQVYENLNKNE       216
AK88         KAVQGSYEETPWVRDVFFKLERNLTQLNEGREEDSLSKKHCYDLNYWLYEQVYENLNKNE       182
PCOAH        EGMKGSYEETPWVRDVFFKLDRNLTEVNENRGEDSLSKKHCYDLNYWLYEQVYENLNKNE       286
C922         EAIEGSYEQTPWVRDVFFKLERNLTEINESREKDSLSKKHCYDLNYWLYEQVYENLNKNE       125
PCYB         EAIEGSYEQTPWVRDVFFKLERNLTELNESRDEDSLSKKHCYDLNYWLYEQVYENLNNNE       108
PcyM         EAIEGSYEQTPWVRDVFFKLERNLTELNESRDEDSLSKKHCYDLNYWLYEQVYENLNNNE       108
PVP01        EAIEGSYEQTPWVRDVFFKLERNLTEINESRGEDSLSKKHCYDLNYWLYEQVYENLNNNE       103
PVX          EAIEGSYEQTPWVRDVFFKLERNLTEINESRGEDSLSKKHCYDLNYWLYEQVYENLNNNE       103
PVL          EAIEGSYEQTPWVRD---------------------------------------------       58
PocGH01      DAIEPSFDRTPWVRDVFFKLERNLTMINENRAEDNFSKKHCYDLNFWLYEQVYKNFESNE       108
PmUG01       DSVEPSFDKTLWARDVFLKLERNVLSVNRNRIEDVFSKKHCYDLNYWLYEQVYKNFDHNE       99
             . ::  :: :  .*:

PCHAS        NSIHFFMTLDILLNSWENMNADQFNGEKDICQPDNTLIDINYLKEIKYLGDYVENFDTIK       155
PCHCB        NSIHFFMTLDILLNSWENMNADQFNGEKDICQPDNTLIDINYLKEIKYLGDYVENFDTIK       155
PBANKA       NSLSFFITLDILLNSWENMNADKFNGNKDICQPDNTLVDINYLKEIKYLADYVENFETIK       155
PY17X        NSLSFFITLDILLNSWENMNADKFNGNKDICQPDNTLVDINYLKEIKYLADYVENFETIK       155
PKNH         NDENFFKIIDDLQGAWKNINNDKFANADNICHPDKTLVDMKYLEDVKDLFDLIEDHSTIK       276
AK88         NDENFFKIIDGLQGAWKNINNDKFPNADNICHPDTTLVDMKYLKDVKYLFDLVEDYSTIK       242
PCOAH        NDENFFKIIDDLQDAWKNINNDKFPNADNICHPDKTLVDMKYLEDVKHLFDFIEDYSTIK       346
C922         NDENFFKIIDGLQDAWKNINNDKFPNAENLCHPDKTLVDMKYLKDVKNLFDFIEDFSTIK       185
PCYB         NDENFFKIIDGLQDVWKNINNDKFPNADNLCHPDKTLIDMKYLKDVKHLFDLIEDFSTIK       168
PcyM         NDENFFKIIDGLQDVWKNINNDKFPNADNLCHPDKTLIDMKYLKDVKHLFDLIEDFSTIK       168
PVP01        NDENFFKIIDGLQNAWTNINNDKFPNADNICHPDKTLVDMKYLKDVKHLFDFIEDFSTIK       163
PVX          NDENFFKIIDGLQNAWTNINNDKFPNADNICHPDKTLVDMKYLKDVKHLFDFIEDFSTIK       163
PVL          -----------------------FPNADNICHPDKTLVDMKYLKDVKHLFDFIEDFSTIK       95
PocGH01      NDENFYKTLDLLIEAWTNINNDKFANEENLCHPDPTLVDMEYLKEIKYLFDFIEDFIIIK       168
PmUG01       KDENFYKTIDIFQDGWKRINNSEFPNEDNVCHPDHTLVDMEYLKDVKNLFDLIEDYVIIK       159
                                    * . .::*:** **:*::**:::* * * :*:.  **

PCHAS        SAAIEDTNKACNVYIDYLRYAIPAYYEWNKLCTLEEENLCNKYIHDYEKYDPKGVLSNLS       215
PCHCB        SAAIEDTNKACNVYIDYLRYAIPAYYEWNKLCTLEEENLCNKYIHDYEKYDPKGVLSNLS       215
PBANKA       SAAIEDTNKACNAYIDYLRSAIPAYYEWNTVCTIEEENICNKYIRDYEKYNPKGVLCNLS       215
PY17X        SAAIEDTNKACNVYIDYLRSAIPAYYEWNTVCTIDEENLCNKYIRDYEKYNPKGVLCNLS       215
PKNH         AAAINDTKNACLKYIDYLKIKVPLYYEWNNICTMEEENICTKYIDDYSKYDPKNVLENLS       336
AK88         AAAINDTKNACQKYIDYLKVKVPLYYEWKDVCTMEEENICTKYIDDFSQYNPKSVLENLS       302
PCOAH        EAAINDTKNACQKYIDYLKITVPLYYEWNGICTMEEQNICTKYIDDYSKYDPKNVMENLS       406
C922         TAAIKDTNRACQKYIDYLKLKVPLYYEWNSVCTMEEENICTKYIDDYPKYNPKNVLENLS       245
PCYB         TAAIKDTNNACQKYIDYLKLKVPLYYEWNDVCTMEEENICTRYIDDYPKYNPKNVLENLS       228
PcyM         TAAIKDTNNACQKYIDYLKLKVPLYYEWNDVCTMEEENICTRYIDDYPKYNPKNVLENLS       228
PVP01        TAAIKDTNNACQKYIDYLKLKVPLYYEWNDVCTMEEENICTKYIDDYPKYNPKNVLENLS       223
PVX          TAAIKDTNNACQKYIDYLKLKVPLYYEWNDVCTMEEENICTKYIDDYPKYNPKNVLENLS       223
PVL          TAAIKDTNNACQKYIDYLKLKVPLYYEWNDVCTMEEENICTKYIDDYPKYNPKDVLENLS       155
PocGH01      TAAIKDTNNACYKYLDYLKKKVPIYYEWKDLCTMEGDNICTRYIDDYVKFNPKNVLENLS       228
PmUG01       AEVIRDTNNACYKYIEYLKQKVPLYYEWENVCTVEEDNICTRYIDDYTKYNPRNVLENLS       219
               .*.**:.**  *::**:  :* ****: :**:: :*:*.:** *: :::*:.*: ***

PCHAS        VTGLAFAQLFNKCYKNIVSIFLSTNNASERTTIKLRNGLETISYGITESQGRSLSEVGIP       275
PCHCB        VTGLAFAQLFNKCYKNIVSIFLSTNNASERTTIKLRNGLETISYGITESQGRSLSEVGIP       275
PBANKA       VTGLAVAQLFNQCYKSIVNIFMNVNNAPVRTTIKLRNGLETISYGITENKGRSLYEVETA       275
PY17X        VTGLAFAQLFNQCYKSIVNIFINVNNAPVRTTIKLRNGLETISYGITENKGRTLSEVETA       275
PKNH         VVSLALASIFNDCYQNIINLFTEAEKIEPRTVLKQRGITGESENNVVKTGGRILAEHVSD       396
AK88         VVSLALASIFNDCYQNIINLFTESEKIEPRTVLKHRDITGQSESNIVKVGGRVLSEAILD       362
```

```
PCOAH        VVSLALASIFNDCYQNIINLFTEAEKIEPRTVLKNRDITGQSENNAIKMGGRILAAAVGD       466
C922         VVSLALSSIFNDCYQDIINLFTKAEKIEPRTVLKVRDITGTSQSNVVKMGGRVLAEAIND       305
PCYB         VVSLALASIFNDCYQNIINLFTEAEKIEPRTVLKHRGITGPSESNVVKIGGRALAEAISD       288
PcyM         VVSLALASIFNDCYQNIINLFTEAEKIEPRTVLKHRGITGPSESNVVKIGGRALAEAISD       288
PVP01        VVSLALASIFNDCYQNIINLFTEAEKIEPRTVLKHRDITGPSESNVVKIGGRALAEAISD       283
PVX          VVSLALASIFNDCYQNIINLFTEAEKIEPRTVLKHRDITGPSESNVVKIGGRALAEAISD       283
PVL          VVSLALASIFNDCYQNIINLFTEAEKIEPRTVLKHRDITGPSESNVVKIGGRALAEAISD       215
PocGH01      VVGLALASIFNDCYQNVINLFTEAEKLPARTLLKHRNSVGKLESTIIKT-GRALAESGSD       287
PmUG01       VVGLSLASVFNDCYQNIITVFQESEKLPSRTELKQRNALGQIESTVVKV-GRTLAEIGDD       278
             *..*:.:.:**.**:.::.:* . ::   ** :* *.     .    :  ** *

PCHAS        IEEKSDFFSPIIYFFNSIYSTISEIYINSYNDIILLIVLFSGILITFFGVNKITKIIKST       335
PCHCB        IEEKSDFFSPIIYFFNSIYSTISEIYINSYNDIILLIVLFSGILITFFGVNKITKIIKST       335
PBANKA       IEEPTDLLAYVIYILKSLYNTISEIYINNYNDIILLIVLFSGILVTCFGVHKITKIVKST       335
PY17X        IQEPADFLAYLIYILKSVYNTISEIYINNYNDIILLIVLFSGILITCFGVYKISKIVKST       335
PKNH         TSESANMLTGVNTLATSPFPMTKRISPCVFNQVVLLVISFLSLLLFVHSIYKFILIGKYI       456
AK88         IPESGNALVGINALATSFFSMFKRFNSYIYSMFAPVGLSLLSIVLFMYVVYKFTPIGKTI       422
PCOAH        TYESGNRLIGINALGTSIFSMVKRFSSYVFSLIAPVGISLLCLLFFLYVLNKFTSIGKYI       526
C922         TSQSGNILIGINALVTSLFSVVKRFNSYVFSLVAPVGVSLLSLFLFLCVLYKFTPIGKSI       365
PCYB         TPQSGNMLIGINALATSLFSVVKRFNSYVFNLVAPVGLSLLSLLLFLYVLYKFTPIGKSI       348
PcyM         TPQSGNMLIGINALATSLFSVVKRFNSYVFNLVAPVGLSLLSLLLFLYVLYKFTPIGKSI       348
PVP01        TSQSGNMLIGINALATSLFSVVKRFNSYVFSLVAPVGLSLLGLLLFLYVLYKFTPIGKSI       343
PVX          TSQSGNMLIGINALATSLFSVVKRFNSYVFSLVAPVGLSLLGLLLFLYVLYKFTPIGKSI       343
PVL          TSQSGNMLIGINALATSLFSVVKRFNSYVFSLVAPVGLSLLGLLLFLYVLYKVSKRMSG-       274
PocGH01      PMPSGSIFVGINAFVSSLFSVMKGFNSYVFNLVTPVSLSLLGILMLFYILYKFSSLRQSI       347
PmUG01       GMQSGDIFIGVNDFVYSLIYAVKRLNSYVFDHFTTMNILLLVILMALFIFCKFISGRSMS       338
                  . :  :  :  *     . :    :.  .  : : :  :..    . *.    .

PCHAS        SNEQNRNQNTREEISL--------------------------------------------       351
PCHCB        SNEQNRNQNTREEISL--------------------------------------------       351
PBANKA       SSVQNRSQNTQAEISL--------------------------------------------       351
PY17X        SNVQNRSQNTQAEIPL--------------------------------------------       351
PKNH         FDTYKGVENKCTRCKSDDSEDDEEEEEEEEEDDEDDDE-------EEEGDEEDEDDEDDD       509
AK88         YLNQKGMKNHYVRKNSDDSDDEDDDDDDDDDNDSANISH------KNKKNNRGNNRDNNR       476
PCOAH        SYTHKGVQNKCLRNKSDDEEDDEEDDDEDDDEDDDDDDDDDDYDYIDEDDDEDDDEDDDD       586
C922         YHPHKRGKNIFVRNKTDDLDDDEDDDDEN------------------------------S       395
PCYB         NLTHKRVKNKFVGNRSDDFDDDEDDDDD--------------------------------       376
PcyM         NLTHKRVKNKFVGNRSDDFDDDEDDDDD--------------------------------       376
PVP01        SRTHKRVKNKFVRNKRDDFDDDEDDD-D--------------------------------       370
PVX          SRTHKRVKNKFVRNKRDDFDDDEDDD-D--------------------------------       370
PVL          ------------------------------------------------------------       274
PocGH01      YRTHKKIKNKFGRNKGKDEIGPLSDCSGSLDSHS--------------------------       381
PmUG01       CNDSKKKK----------------------------------------------------       346


PCHAS        -----------------------    351
PCHCB        -----------------------    351
PBANKA       -----------------------    351
PY17X        -----------------------    351
PKNH         DDDSDSNSALKSSFRKWSLNSHS    532
AK88         DNNRDNNKDNNSSNVE-------    492
PCOAH        EDDSNNNSGLKSSSSEEPLNSHS    609
C922         DNNTDNNSDFKSSPSIESLNSHS    418
PCYB         -DDSDNNSDLKSSSSMESLNSHS    398
PcyM         -DDSDNNSDLKSSSSMESLNSHS    398
PVP01        -DDSDNSSDLKSSSSMESLLSNS    392
PVX          -DDSDNSSDLKSSSSMESLLSNS    392
PVL          -----------------------    274
PocGH01      -----------------------    381
PmUG01       -----------------------    346
```

</pre></body></html>
